# Artificial Intelligence Decision-Making Transparency and Employees’ Trust: The Parallel Multiple Mediating Effect of Effectiveness and Discomfort

**DOI:** 10.3390/bs12050127

**Published:** 2022-04-27

**Authors:** Liangru Yu, Yi Li

**Affiliations:** School of Economics and Management, Chongqing University of Posts and Telecommunications, Chongqing 400065, China; s200701038@stu.cqupt.edu.cn

**Keywords:** AI decision-making transparency, trust, effectiveness, discomfort

## Abstract

The purpose of this paper is to investigate how Artificial Intelligence (AI) decision-making transparency affects humans’ trust in AI. Previous studies have shown inconsistent conclusions about the relationship between AI transparency and humans’ trust in AI (i.e., a positive correlation, non-correlation, or an inverted U-shaped relationship). Based on the stimulus-organism-response (SOR) model, algorithmic reductionism, and social identity theory, this paper explores the impact of AI decision-making transparency on humans’ trust in AI from cognitive and emotional perspectives. A total of 235 participants with previous work experience were recruited online to complete the experimental vignette. The results showed that employees’ perceived transparency, employees’ perceived effectiveness of AI, and employees’ discomfort with AI played mediating roles in the relationship between AI decision-making transparency and employees’ trust in AI. Specifically, AI decision-making transparency (vs. non-transparency) led to higher perceived transparency, which in turn increased both effectiveness (which promoted trust) and discomfort (which inhibited trust). This parallel multiple mediating effect can partly explain the inconsistent findings in previous studies on the relationship between AI transparency and humans’ trust in AI. This research has practical significance because it puts forward suggestions for enterprises to improve employees’ trust in AI, so that employees can better collaborate with AI.

## 1. Introduction

Artificial Intelligence (AI) is a new generation of technology that can interact with the environment and aims to simulate human intelligence [1]. In recent years, more and more enterprises have introduced AI, and how to encourage employees to accept AI, use AI, and trust AI has become a hot research topic. Whether AI can be successfully integrated into enterprises and serve as a decision maker depends crucially on employees’ trust in AI [1,2]. Humans’ trust in AI refers to the degree to which humans consider AI to be trustworthy [2]. Transparency, which reflects the degree to which humans understand the inner workings or logic of a technology, is essential for building trust in new technologies [3]. Transparency is more problematic for AI than for other technologies [1]. The operation process of AI (usually based on deep learning methods) is complex and multi-layered, and the logic behind it is difficult to understand [1]. As a result, AI’s decision-making process is considered non-transparent [1]. The relationship between AI transparency and trust, and how AI transparency affects trust, is unclear [4].

Many previous studies have explored the relationship between AI system transparency and humans’ trust in AI, with inconsistent conclusions. First, some studies have found a positive correlation. For example, the transparency of music recommendation systems promotes user trust [5,6]. Providing explanations for automated collaborative filtering systems can increase users’ acceptance of the systems [7], and providing explanations for recommendation systems can increase users’ trust in the systems [8]. 

However, some studies found no correlation. For example, Cramer et al.’s [9] study on recommendation systems in the field of cultural heritage did not find a positive effect of transparency on trust in the systems, although transparency did increase acceptance of recommendations. Meanwhile, Kim and Hinds [10] investigated the effect of robot transparency on trust and blame attribution and found no significant effects. 

FInally, some studies have found an inverted U-shaped relationship. For example, advertisers develop algorithms to select the most relevant advertisements for users, but an appropriate level of transparency of advertising algorithms is needed to enhance trust and satisfaction [11]. Too vague or too specific explanations in advertisements will produce feelings of anxiety and distrust, whereas moderate explanations in advertisements enhance trust and satisfaction [11]. Kizilcec [12] found that providing students with high or low levels of transparency is detrimental, as both extremes confuse students and reduce their trust in a system. In other words, providing some transparent information helps promote trust, whereas providing too much or too little information may counteract this effect. The above research shows that the relationship between AI transparency and trust is inconsistent, and more research is needed to explore it. 

In terms of how AI transparency affects human trust in AI, research on the mediating mechanism between the two is relatively lacking. Zhao et al. [13] investigated whether providing information about how online-shopping advice-giving systems (AGSS) work can enhance users’ trust, and they found that users’ perceived understanding of AGSS plays a mediating role between subjective AGS transparency and users’ trust in AGSS. Cramer et al. [9] studied the influence of transparency of recommendation systems on users’ trust and took perceived competent and perceived understanding as mediating variables; they found that the transparent versions of recommendation systems would be easier to understand but would not be considered as more competent. 

This paper finds that previous studies on the mediating variables are scarce and lacking from the perspective of emotion, which makes it difficult to clearly explain the effect of AI transparency on humans’ trust in AI.

In summary, this paper builds a model that contains a parallel multiple mediating effect—that is, one mediating path has a positive effect on trust, and another mediating path has a negative effect on trust. This paper focuses on the human–AI collaborative work scenario, wherein AI is the primary decision maker, and studies how AI decision-making transparency (non-transparency vs. transparency) affects employees’ trust in AI. 

Trust is influenced by cognitive and emotional aspects [1]. On the cognitive side, high efficiency is a characteristic of AI [14], and human perception of AI performance affects human trust in AI [15]. On the emotional side, discomfort affects people’s emotional response to new technologies [16]. Based on the research of Castelo et al. [15], this study selected effectiveness and discomfort as cognitive and emotional variables and tested their mediating effects.

The paper makes three key contributions. First, the discovery of the parallel multiple mediating effect provides a partial explanation for the inconsistent relationship between AI transparency and humans’ trust in AI found in previous studies. Specifically, perceived transparency increases trust by increasing effectiveness, while it reduces trust by increasing discomfort. Second, we find one mediating path and two chain mediating paths to explain the mediating effect between AI transparency and employees’ trust in AI. Specifically, one mediating path and two chain mediating paths exist between AI decision-making transparency and employees’ trust in AI: the mediating effect of employees’ perceived transparency, the chain mediating effect of employees’ perceived transparency and the perceived effectiveness of AI, and the chain mediating effect of employees’ perceived transparency and their discomfort with AI. Third, effectiveness and discomfort are selected from cognitive and emotional perspectives to construct cognitive and emotional pathways. This is a pioneer study that examine the effects of AI transparency on humans’ trust in AI from an emotional perspective. Therefore, this study’s significance is to help people understand how AI decision-making transparency affects employees’ trust in AI in human–AI collaborative teams where AI is the decision maker, and to provide enterprises with advice on improving employees’ trust in AI.

## 2. Background Literature and Research Hypotheses

### 2.1. AI in Enterprises Requires Trust

The application of AI to enterprises can generate a great deal of value and greatly improve the efficiency and effectiveness of enterprises [17]. For example, AI can improve the accuracy of recommendation systems and increase the confidence of users [18]. AI is beneficial to performance management, employee measurement, and evaluation in enterprises [19]. AI can enhance human capabilities by making decisions in the enterprise [20]. AI in the enterprise reduces potential conflict by standardizing decision-making procedures, thereby reducing pressure on supervisors and team leaders [21].

However, whether AI can be successfully integrated into enterprises and become the main decision maker depends critically on employees’ trust in AI [1]. First, AI as a decision maker has the power to make decisions that are very relevant to employees and that influence employees [22,23]. Therefore, trust in the context of AI decision-making is necessary, and influences employees’ willingness to accept and follow AI decisions; trust may potentially promote further behavioral outcomes and attitudes related to the validity of AI decisions [2]. In addition, when AI is the primary decision maker, lack of trust negatively affects human–AI collaboration in multiple ways. One reason is that lack of trust can lead to brittleness in the design and use of decision support systems. If the brittleness of a system leads to poor recommendations, it is likely to strongly influence people to make bad decisions [24]. Another reason is that high-trust teams generate less uncertainty, and problems are solved more efficiently [25]. Further, if employees do not believe in AI, enterprises or organizations may not be able to apply AI because of trust issues. For example, lack of trust is an important factor in the failure of sharing economy platforms [26]. Therefore, trust can enhance human–AI collaboration in an enterprise. In order for AI to make better decisions, AI requires trust.

### 2.2. SOR Model

Mehrabian and Russell [27] first proposed the stimulus–organism–response (SOR) theory, confirming that when an individual is incited by external stimuli (S), certain internal and physical states (O) will be generated, and then an individual response (R) will be triggered. External stimuli trigger an individual’s internal state, which can be either a cognitive state or an emotional state, and then the individual decides what action to take [28]. SOR models have been used in AI scenarios. Xu et al. [29] studied the influence of a specific design of a recommendation agent interface on decision making, taking trade-off transparency as an external stimulus in the SOR model and measuring trade-off transparency at different levels. Saßmannshausen et al. [30] used the SOR model to study humans’ trust in AI, where external characteristics were the stimuli, the perception of AI characteristics was the individual internal state, and trust in AI was the individual response.

In sum, the SOR model has been used in AI scenarios in previous studies, with transparency as the external stimulus and trust in AI as the individual response. This paper argues that AI decision-making transparency is an external stimulus that conveys decision-making information to employees. AI decision-making transparency can lead not only to cognitive states but also to emotional states in employees. The cognitive states caused by transparency include perceived competence [9] and perceived understanding [13], among others. There are few studies on emotional states caused by transparency. Eslami et al. [11] believed that including overly specific and general explanations would make people feel “creepy”. An employee’s perceived transparency is the employee’s cognitive state in relation to an external transparency stimulus [13]; effectiveness and discomfort are an employee’s internal cognitive and emotional states [15]; and trust is an employee’s response.

This study selects effectiveness and discomfort as employees’ cognitive and emotional states, based on the research of Castelo et al. [15]. According to the SOR model, when employees are stimulated by AI decision-making transparency, they first generate a cognitive state (employees’ perceived transparency) in relation to the external transparency stimulus, and then they generate their internal cognitive states (employees’ perceived AI effectiveness) and emotional states (employees’ discomfort with AI), which finally trigger employees’ trust in AI. Therefore, the cognitive path is AI decision-making transparency → employees’ perceived transparency → employees’ perceived AI effectiveness → employees’ trust in AI. On the other hand, the emotional path is AI decision-making transparency → employees’ perceived transparency → employees’ discomfort with AI → employees’ trust in AI.

### 2.3. Algorithmic Reductionism

According to algorithmic reductionism, the quantitative characteristics of algorithmic decision making will cause individuals to perceive the decision-making process as reductionist and decontextualized [31]. For example, Nobel et al. [32] found that candidates believed AI could not “read between the lines”. Although current algorithms are considered to be highly efficient [14], algorithmic reductionism refers to how people affected by an algorithm’s decisions subjectively perceive the decision-making process, independent of the algorithm’s objective validity [31]. Existing studies have found that individuals believe that AI decision-making results are obtained by statistical fitting based on limited data [33]. Therefore, individuals think that AI decision-making ignores background and environmental knowledge [33], thereby simplifying information processing. Therefore, algorithmic reductionism is mainly used to explain the individual’s perception of and feelings about the AI decision-making process. Employees will think of AI decision-making process as reductionistic, especially for non-transparent decision-making.

### 2.4. Social Identity Theory

Social identity theory believes that individuals identify with their own groups through social classification and generate in-group preferences and out-group biases [34]. In addition, people like to believe that their inner group is unique, and when the outer group begins to challenge this uniqueness, the outer group will be judged negatively [35]. Negative emotions toward AI occurs when employees realize that AI is becoming more and more human-like and beginning to challenge the uniqueness of human work.

### 2.5. AI Decision-Making Transparency and Employees’ Perceived Transparency

In an organizational context, transparency refers to the availability of information about how and why an organization or other entity makes decisions [36]. Decision making is divided into three levels [36]: (1) non-transparency (the final decision is simply announced to the participants); (2) transparency in rationale (the final decision and the reasons for it are announced to the participants); and (3) transparency in process (the final decision and reasons are announced and the participants have an opportunity to observe and discuss the decision-making process) [36]. In the AI context, de Fine Licht et al. [37] stated that a transparent AI decision-making process includes goalsetting, coding, and implementation stages. Referencing earlier studies on transparency and AI decision-making transparency, this paper defines AI decision-making non-transparency as informing employees only of the AI decision-making results, whereas AI decision-making transparency is defined as informing employees of the AI decision-making result, rationale, and process [36,37].

AI decision-making transparency is thus the degree to which an AI system releases objective information about its working mode [13], whereas employees’ perceived transparency refers to the availability of employees’ subjectively perceived information [13]. Thus, AI decision-making transparency (i.e., objective transparency) and employees’ perceived transparency (i.e., subjective transparency) are different. Zhao et al. [13] proved that objective transparency has a positive effect on subjective transparency. If an AI system provides more information (objective transparency), employees receive more information (subjective transparency); that is, more AI decision-making transparency will lead to an increase in employees’ perceived transparency [13].

Moreover, people prefer AI decision-making transparency to non-transparency for several reasons: (1) limited transparency is used as a common technique to hide the interest-related information of the real stakeholders, which can be avoided by full transparency [38]; (2) transparency increases the public’s understanding of decision making and the decision-making process, thereby making the public more confident in decision makers [37]; (3) transparency has positive results, including increasing legitimacy, promoting accountability, supporting autonomy, and increasing the principal’s control over the agent [36,39,40,41]; and (4) transparency is a means to overcome information asymmetry [42] and to make the public believe that the decision-making process is fair [37]. Therefore, people subjectively prefer that more information be disclosed. The more AI decision-making transparency, the better people feel subjectively. The more information AI provides, the more useful information people are likely to receive from it; that is, the subjective transparency is improved [13]. Hence, this paper argues that AI decision-making transparency leads to greater perceived transparency, compared with AI decision-making non-transparency, in the human–AI collaborative work scenario where AI is the primary decision-maker. 

Therefore, we hypothesize:

**Hypothesis** **1** **(H1).** *AI decision-making transparency (vs. non-transparency) leads to greater employees’ perceived transparency.*

### 2.6. Mediating Role of Employees’ Perceived Transparency between AI Decision-Making Transparency and Employees’ Trust in AI

A large number of studies have shown that providing objective transparency can enhance users’ trust in AI [2,43,44]. However, there may be deficiencies in this view. We believe that objective transparency does not directly affect employees’ trust in AI; rather, objective transparency should first affect subjective transparency. Conversely, employees’ trust in AI should be directly affected by employees’ perception of available information; that is, subjective transparency (employees’ perceived transparency) directly affects trust. The reasons for this are as follows. First, after employees receive the information provided by the AI system, the first step is to make sure that the information is available—that is, to ensure subjective transparency (employees’ perceived transparency) [13]. This paper argues that AI decision-making transparency (vs. non-transparency) leads to greater employees’ perceived transparency. Second, greater employees’ perceived transparency indicates that more information is disclosed [37]. The higher the level of information disclosure, the higher will be the level of trust [45]. In general, AI decision-making transparency increases employees’ trust in AI by increasing their perceived transparency. Therefore, we hypothesize:

**Hypothesis** **2** **(H2).** *Employees’ perceived transparency mediates the impact of AI decision-making transparency on employees’ trust in AI.*

### 2.7. Chain Mediating Role of Employees’ Perceived Transparency and Employees’ Perceived Effectiveness of AI

Employees’ perceived effectiveness of AI refers to employees’ belief in the performance of AI as the primary decision maker [15]. The fact that algorithmic judgments have higher accuracy than human judgments has been proven by many scholars and is widely accepted [46]. AI has strong cognitive ability and can convincingly demonstrate that ability [47]. The more powerful that employees perceive AI’s cognitive ability to be, the more they will perceive the effectiveness of AI and thus trust AI, and vice versa. Therefore, we hypothesize:

**Hypothesis** **3** **(H3a).** *Employees’ perceived effectiveness of AI has a positive impact on employees’ trust in AI.*

According to algorithmic reductionism, employees believe that the AI decision-making process is reductionist [31]. The perceived reductionist nature of the AI decision-making process prevents interactive employees from appreciating the high performance of AI, while increased explanations of decisions can restore employees’ faith in the AI decision-making process and offset the impact of the reductionist process. In addition, Zhang et al. [48] found that revealing AI performance information can improve people’s trust in AI output. In sum, we believe that when AI decision-making transparency increases, employees perceive more available information. A large amount of detailed information reveals the excellent performance and strong reliability of AI. Employees will perceive the high performance of AI in decision-making from a large amount of information, thereby enhancing their belief in AI performance (i.e., perceived effectiveness of AI). In the end, employees will develop higher trust in AI. Therefore, we hypothesize:

**Hypothesis** **3** **(H3b).** *Employees’ perceived transparency and perceived effectiveness of AI have a chain mediating role between AI decision-making transparency and employees’ trust in AI.*

### 2.8. Chain Mediating Role of Employees’ Perceived Transparency and Employees’ Discomfort with AI

Employees’ discomfort with AI refers to the discomfort of employees caused by the use of AI [15]. In this study, it refers specifically to discomfort with AI decision making. Employees may not trust AI because they feel that AI lacks emotion. For example, Ryan [47] argued that AI cannot be trusted because it does not have emotional states. The more AI’s lack of emotional ability is perceived by employees, the more they will feel uncomfortable with AI, and thus the less trust they will have in AI. Conversely, the less AI’s lack of emotional ability is perceived by employees, the less discomfort they will feel with AI, and thus the more trust they will have in AI. Therefore, we hypothesize:

**Hypothesis** **4** **(H4a).** *Employees’ discomfort with AI has a negative impact on employees’ trust in AI.*

According to social identity theory, when an out-group threatens the uniqueness of an in-group, in-group members will react negatively to the out-group [34]. Transparency and understandability are important factors that make AI algorithms human-like [49]. Increasing the transparency of AI decision making and enabling employees to understand the algorithms are steps that can make employees feel that AI is more human-like. However, human-like AI challenges the uniqueness of human employees, and human employees may have negative reactions to AI, such as fear that AI will replace human jobs [50]. Castelo et al. [15] used social identity theory to argue that as algorithms become increasingly human-like, people may feel increasingly uncomfortable with the use of such algorithms. Discomfort hinders the acceptance and use of new technology [16,51]. Therefore, this study assumes that AI decision-making transparency will make AI human-like. When employees find AI decision making to be more human-like, they will be more uncomfortable with and distrustful toward AI. Therefore, we hypothesize:

**Hypothesis** **4** **(H4b).** *Employees’ perceived transparency of and discomfort toward AI have a chain mediating role between AI decision-making transparency and employees’ trust in AI.*

**Figure 1:**
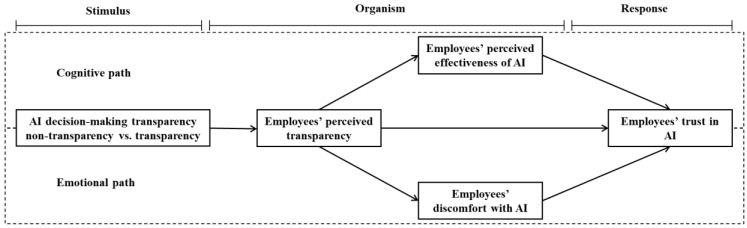
Research model.

The research model for this study is summarized in Figure 1.

## 3. Methods

We used the experimental vignette methodology (i.e., presenting a scenario in a written text). Participants were randomly assigned to one of two conditions (AI decision-making transparency: non-transparency vs. transparency). The vignette describes the human–AI collaborative work scenario, where AI is the primary decision maker on task assignment. The reasons for choosing this vignette are as follows: (1) this vignette (scenario of the lighting system team at company Car Solutions) refers to Ötting and Maier’s work [21], which has been shown to be effective; (2) task assignment occurs in daily work and may be made by AI; and (3) the vignette describes a mixed team that can embody human–AI collaboration. In addition, we added scene pictures and scene background descriptions to help participants imagine themselves in the scene as much as possible.

This study was conducted in accordance with the Personal Information Protection Law of the People’s Republic of China, and the research proposal was approved by the Ethics Committee of Chongqing University of Posts and Telecommunications (Identification Number: 2022-0002). All participants gave informed consent for inclusion before they participated in the study.

### 3.1. Sample and Data Collection

We recruited 301 participants (143 males, 158 females) with work experience from Tencent Questionnaires (a Chinese questionnaire survey system similar to Mturk; papers based on the data collected by Tencent Questionnaires have been accepted by journals [52,53,54]) to complete the online questionnaire. The sample database of Tencent Questionnaires currently exceeds 1 million people. The platform sent our recruitment information to the participants who met the requirements. The participants decided whether or not to participate in the survey. After reaching a certain number, the platform stopped recruiting. After a participant clicked a provided link, a vignette would be randomly displayed, and two groups (153 non-transparency vs. 148 transparency) were randomly assigned. 

The sample was strictly selected. First, participants with work experience were selected through the participant selection function provided by Tencent Questionnaires. Second, we set up screening questions in the questionnaire to remove participants who were not serious in filling out the questionnaire. Third, we strictly screened the questionnaires and eliminated those with abnormal information, logical errors, regular answers, or overly short response times. Finally, in the questionnaire, the item “Can you feel you are immersed in the scene?” (1 = cannot feel it at all, 7 = can feel it completely) scored 5.26, and the item “Do you think the above scenario is easy to understand?” (1 = cannot understand it at all, 7 = can understand it completely) scored 5.33, which are greater than the median value of 4, indicating that participants imagined themselves in the experimental scenario and were able to understand it. 

After screening, only 235 people (113 males vs. 122 females; 115 non-transparency vs. 110 transparency) completed the questionnaire effectively. Sample size was determined by prior power analysis, performed using G*Power, with the following settings: two-tailed, effect size: 0.5, alpha: 0.05, and power: 0.95. The sample size of each group calculated by G * Power was 105, and the sample size of this study met the requirements. The sample characteristics were as follows: the participants were under 45 years of age (M = 28.29, SD = 2.94); in terms of monthly income, the mean value was 6901.70 yuan, and the standard deviation was 3512.74 yuan. The highest degree of most participants was a bachelor’s degree (53.62%). Participants came from various industries, with the largest proportion from production/processing/manufacturing (27.66%), IT/communication/electronics/Internet (23.83%), and hotel/catering/tourism/medical/healthcare (11.91%), and the rest from professional services/consulting (finance/accounting/human resources, etc.) (9.79%), the financial industry (5.53%), traffic/transportation/logistics/warehousing (4.68%), civil servant/public institutions (3.83%), and others (12.77%). All participants had work experience.

### 3.2. Procedure and Manipulation

Participants received the link from the Tencent Questionnaire. After opening the link, they were notified that this survey was completely anonymous, the provided information would be kept strictly confidential, and all information would be used only for academic research. Next, participants were randomly assigned to one of two experimental vignettes (non-transparency vs. transparency) (see Appendix A for the complete vignette). 

**Figure A1:**
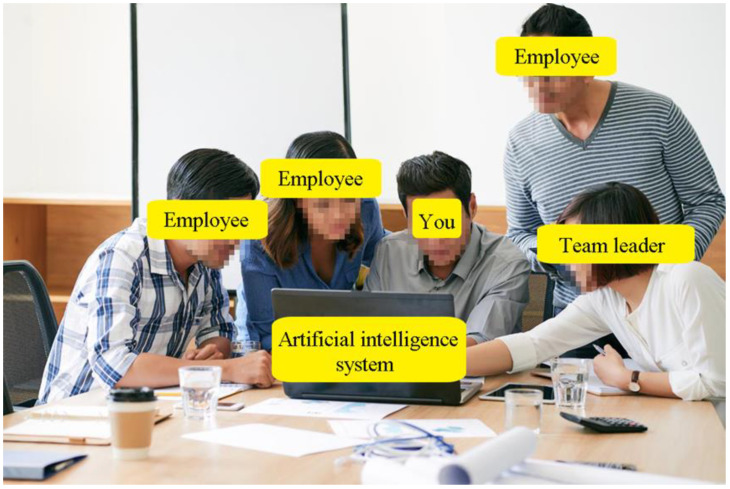
Employees interacting with the AI system used in study.

The vignette described the daily condition of employees of an automobile company. The employee worked in a mixed team consisting of three other employees, a team leader, and an AI system. The decision maker was the AI system, which made the decisions on task assignment. This experimental vignette referred to Ötting and Maier [21]. We included a picture of employees interacting with the AI system (see Appendix B, Figure A1) to help the participants better understand the experimental vignette. AI decision-making transparency was manipulated by varying the AI decision-making result, rationale, and process. This kind of manipulation is a common practice when using an experimental vignette in transparency research [36,41].

### 3.3. Measures

This study used four key constructs: perceived transparency, effectiveness, discomfort, and trust. All four constructs were measured using seven-point Likert scales (1 = very inconsistent; 7 = very consistent). The scale of perceived transparency came from Zhao et al. [13], the scale of effectiveness and discomfort came from Castelo et al. [15], and the scale of trust came from Höddinghaus et al. [2].

Since all participants were Chinese, we translated the scale from English to Chinese and made appropriate modifications according to the situation. For example, we changed an item in the perceived transparency scale from “I can get a lot of information to understand how the system works” to “how the AI system works” to suit our context. The measurement items of each variable are shown in Table 1. To ensure the accuracy of scale translation and revision, we first invited a professor who studies AI and management to review the revised content, and we then invited a bilingual professor specializing in organizational behavior to check the Chinese and English scales.

### 3.4. Data Analysis

This study used SPSS 23 (IBM Corp: Armonk, NY, USA) to test the reliability of the data and LISREL 8.80 (Scientific Software International, Inc.: Lincolnwood, IL, USA) to test the validity of the data. One-way ANOVA was used to test the difference between non-transparency and transparency. LISREL 8.80 was used to establish a structural equation model to test the significance of the model path. The study used the bootstrapping method with SPSS PROCESS macro (Model 81) to test the mediating effect [55].

## 4. Results

### 4.1. Validity and Reliability

The Cronbach’s α values of perceived transparency, effectiveness, discomfort, and trust were 0.851, 0.805, 0.897, and 0.867, respectively. The study then used LISREL 8.80 to test the validity of the data. The confirmatory factor analysis (CFA) results showed that the four-factor (trust, effectiveness, discomfort, perceived transparency) model fitted well based on the following fit statistics: χ2 = 59.426, df = 48, χ2/df = 1.238, RMSEA = 0.0421, NNFI = 0.988, CFI = 0.991, IFI = 0.992, GFI = 0.931, AGFI = 0.888. In contrast, the substituted Haman single-factor model fitted poorly: χ2 = 914.151, df = 54, χ2/df = 16.929, RMSEA = 0.261, NNFI = 0.558, CFI = 0.638, IFI = 0.640, GFI = 0.606, AGFI = 0.430. This showed that the homology error was unlikely to be a serious issue. The standardized factor loadings of each item were between 0.706 and 0.949 and all reached a high level of significance (*p* < 0.001) with good convergent validity. The results of the confirmatory factor analysis and reliability analysis are shown in Table 2.

The composite reliability (CR) of each variable was above 0.70, and the Cronbach’s α coefficient was also above 0.70. This showed that the internal consistency of each variable scale was relatively strong and had high stability and credibility. The average of each variable was above 0.50, indicating that the measurement variable scale had convergent validity.

Table 3 shows the mean value, standard deviation, Pearson correlation coefficient, and square root of the average of all variables in this study. All variables were significantly correlated. Effectiveness, perceived transparency, and trust (r = 0.699, 0.382, *p* < 0.01) were significantly positively correlated, and discomfort was significantly negatively correlated with trust (r = −0.260, *p* < 0.01). Perceived transparency was significantly positively correlated with effectiveness and discomfort (r = 0.353, 0.172, *p* < 0.01). The square root of the average of each variable was between 0.774 and 0.868, which was greater than the correlation coefficient between the variables, indicating that the questionnaire scale had high discriminant validity.

### 4.2. The Results of Variance Analysis of AI Decision-Making Non-Transparency and AI Decision-Making Transparency

The results of the one-way analysis of variance showed that, in terms of perceived transparency, the scores of the participants in the transparency group were significantly higher than those in the non-transparency group. The mean value of the non-transparency group was 4.470 and the mean value of the transparent group was 5.014, F (1, 123) = 11.336 (*p* < 0.01). This showed that this study successfully manipulated the two types of AI decision transparency, and H1 was supported.

### 4.3. Hypotheses Test

The SPSS PROCESS macro (Model 81) [55] was used to test the mediating effect of effectiveness and discomfort, with AI decision-making transparency (non-transparency = 0, transparency = 1) as the independent variable, trust as the dependent variable, and perceived transparency, effectiveness, and discomfort as the mediating variables (the results are shown in Table 4 and Table 5). The bootstrapped sample was 5000. As shown in Table 4, AI decision-making transparency had a significant positive impact on perceived transparency (β = 0.544, *p* < 0.001). Perceived transparency had a significant positive impact on effectiveness, discomfort, and trust (β = 0.277, *p* < 0.001; β = 0.184, *p* < 0.01; β = 0.203, *p* < 0.001). Effectiveness had a significant positive impact on trust (β = 0.734, *p* < 0.001), so H3a was supported. Discomfort had a significant negative impact on trust (β = −0.146, *p* < 0.01), so H4a was supported.

The analysis results in Table 5 showed that the total effect of AI decision-making transparency on trust was 0.086, and the confidence interval was [−0.229, 0.401], including 0, which was not significant. The direct effect of AI decision-making transparency on trust was −0.119, and the confidence interval was [−0.341, 0.104], including 0, which was not significant. The mediating effect discussed in this study mainly includes three indirect effects: Indirect effect 1, AI decision-making transparency → perceived transparency → trust (ADT → EPT → ETA); the confidence interval was [0.034, 0.212] and did not include 0, and thus the mediating effect of perceived transparency was significant. Indirect effect 2, AI decision-making transparency → perceived transparency → effectiveness → trust (ADT → EPT → EPE → ETA); the confidence interval was [0.037, 0.210] and did not include 0, and thus the chain mediating effect of perceived transparency and effectiveness was significant. Indirect effect 3, AI decision-making transparency → perceived transparency → discomfort → trust (ADT → EPT → EDA → ETA); the confidence interval was [−0.075, −0.002] and did not include 0, and thus the chain mediating effect of perceived transparency and discomfort was significant. Therefore, H2, H3b, and H4b were supported.

## 5. Discussion

First, AI decision-making transparency (vs. AI decision-making non-transparency) leads to more employees’ perceived transparency; that is, AI decision-making transparency is positively translated to employees’ perceived transparency. This is consistent with existing research results [13].

Second, this paper confirmed the parallel multiple mediating effect; that is, one mediating path has a positive effect on trust, and another has a negative effect on trust. Employees’ perceived effectiveness of AI has a positive impact on employees’ trust in AI (H3a). Employees’ discomfort with AI has a negative impact on employees’ trust in AI (H4a). This is consistent with the findings of Castelo et al. [15] that effectiveness is positively correlated with reliance on algorithms, and discomfort is negatively correlated with reliance on algorithms. Employees’ perceived transparency and employees’ perceived effectiveness of AI have a chain mediating role between AI decision-making transparency and employees’ trust in AI (H3b). Employees’ perceived transparency and employees’ discomfort with AI have a chain mediating role between AI decision-making transparency and employees’ trust in AI. (H4b). In the context of the present research, employees’ perceived transparency positively affects employees’ trust in AI; however, we can speculate that in other scenarios, the inhibition of trust by discomfort may be equal to or greater than the promotion of trust by effectiveness, leading to different results.

Third, this paper found that employees’ perceived transparency mediates the impact of AI decision-making transparency on employees’ trust in AI (H2); that is, AI decision-making transparency (vs. non-transparency) increases employees’ trust in AI by generating greater employees’ perceived transparency. This is partly consistent with the results of previous studies, which proved that objective transparency has a positive impact on trust [6,56], or that perceived transparency has a positive effect on trust [57].

### 5.1. Theoretical Implications

The theoretical implications of this study are as follows. First, previous studies have reached inconsistent conclusions on the relationship between AI transparency and humans’ trust in AI. This paper finds a parallel multiple mediating effect; that is, perceived transparency enhances trust by increasing effectiveness and, at the same time, reduces trust by increasing discomfort, which partly explains the inconsistent conclusions of previous studies. Second, this study fills the gap created by previous studies’ lack of exploration of the mediating mechanism between AI transparency and humans’ trust in AI. In this paper, we found a mediating path and two chain mediating paths between AI decision transparency and employees’ trust in AI. We found that perceived transparency plays an important mediating role, because three mediating paths pass through perceived transparency. Third, most of the previous literature studied the influence of transparency on human’s trust in AI from the cognitive perspective, while research from the emotional perspective was still lacking [1]. From the cognitive and emotional perspectives, this paper enriches the research on the impact of transparency on people’s trust in AI from the emotional perspective. Fourth, previous studies mostly regarded AI transparency as a variable in studying the relationship between AI transparency and humans’ trust in AI [2,43]. In this paper, AI decision-making transparency was divided into non-transparent and transparent, and an experimental vignette was used to study the transmission between AI decision-making transparency (objective) and employees’ perceived transparency (subjective). 

### 5.2. Practical Implications

The practical implications of this research are as follows. In human–AI collaboration organizations, whether AI can successfully integrate into organizations and serve in a decision-making role depends on employees’ trust in AI [1]. However, the impact of AI transparency on humans’ trust in AI is complex. When improving AI decision-making transparency, it is necessary to improve employees’ perceived transparency and employees’ perceived effectiveness of AI, as well as to avoid employees’ discomfort with AI, so as to promote employees’ trust in AI. Therefore, organizations should take measures from the following three principles: First, organizations should increase AI decision-making transparency by publicly informing employees of the results of AI decision-making, the rationales for AI decision-making, and the process of AI decision making [36]. For the new generation of employees born in the Internet age, transparency is appreciated [58]. Second, organizations should improve employees’ perceived transparency through AI-related training, which will help employees to understand the operation of AI, making it easier for them to understand and receive information transmitted by AI [13]. Third, organizations should let employees use AI to increase the involvement of AI in the enterprise. When employees use AI, they will see the effectiveness, performance, and accuracy of AI. On the other hand, AI lacks emotional ability [47]; therefore, organizations should allow AI to participate in more objective (vs. subjective) tasks, because subjective (vs. objective) tasks require more emotional ability [15], which cannot reflect the efficiency of AI. Finally, increasing people’s trust in AI has social value. Because AI creates multiple opportunities for individual well-being and the prosperity and advancement of individuals, organizations, and societies, trustworthy AI is the foundation of societies, economies, and sustainable development [59].

### 5.3. Limitations and Suggestions for Future Research

Several limitations of this study need to be addressed. First, this research used an experimental vignette methodology, which described a situation similar to one in the real world. Although we provided a picture to help participants become more immersed in the scenario, we could not fully elicit the true psychological reaction of the participants with this method, which limited the external validity. Second, the experimental vignette used in this paper was based on the study of Ötting and Maier [21], which was conducted in Germany, whereas the present study was conducted in China. Due to differences in culture and actual organizational conditions, Chinese participants may potentially have faced difficulty in understanding the scenario. Future research should repeat the experiment in other countries with different cultural backgrounds to ensure the validity of the scenario. Third, this experimental vignette only addressed the decision of task assignment. Since organizational decision making is diverse, future research should investigate different types of decisions. Fourth, this study did not look for boundary conditions. Future studies should investigate more moderating variables, identify limitations and scope of application, and seek mitigation measures. Fifth, the variable measurement in this study adopted the form of a self-reported questionnaire, and the data source was single, so the questionnaire may have been affected by common method bias. Future research could conduct laboratory studies that allow participants to experience real-world situations. Sixth, control variables were not considered in our model. Future research could carry out more detailed research, using employees’ technical knowledge, self-efficacy beliefs, and other factors as control variables.

## 6. Conclusions

This paper aimed to explore how AI transparency affects humans’ trust in AI. Based on the SOR model, algorithmic reductionism, and social identity theory, we constructed a research model to examine how AI decision-making transparency affects employees’ perceived transparency, employees’ perceived effectiveness of AI, employees’ discomfort with AI, and the subsequent effects of employees’ trust in AI. Using the experimental vignette methodology, we simulated a human–AI collaborative work scenario with AI as the primary decision-maker and recruited 235 people to complete the online experiment. After empirical testing, we found that AI decision-making transparency (vs. non-transparency) led to higher perceived transparency, which in turn increased both effectiveness (which promoted trust) and discomfort (which inhibited trust).

## Figures and Tables

**Table 1:** Measurement items of each variable.

Construct	Items	References
Perceived transparency	I can access a great deal of information which explaining how the AI system works.	Zhao et al. [13]
	I can see plenty of information about the AI system’s inner logic.	
	I feel that the amount of the available information regarding the AI system’s reasoning is large.	
Effectiveness	I think AI system makes better decision than human.	Castelo et al. [15]
	I think the decisions made by AI system is useful.	
	I think AI system can make decision very well.	
Discomfort	The decision made by the AI system makes me feel uncomfortable.	Castelo et al. [15]
	The decision made by the AI system makes me feel resistant.	
	The decision made by the AI system makes me feel unsettled.	
Trust	I would heavily rely on AI system.	Höddinghaus et al. [2]
	I would trust AI system completely.	
	I would feel comfortable relying on AI system.	

**Table 2:** Results of reliability and validity analysis.

Factors	Items	Standardized Factor Loadings (λ)	T-Value	Residual Variance (1–λ^2^)	Cronbach’s α	Composite Reliability (CR)	Average Variance Extracted (AVE)
Trust	TRU01	0.801	10.789	0.358	0.867	0.872	0.695
	TRU02	0.904	12.932	0.183			
	TRU03	0.792	10.611	0.373			
Effectiveness	EFF01	0.706	8.803	0.502	0.805	0.817	0.599
	EFF02	0.800	10.422	0.360			
	EFF03	0.812	10.645	0.341			
Discomfort	DIS01	0.850	11.827	0.278	0.897	0.901	0.753
	DIS02	0.949	14.018	0.099			
	DIS03	0.798	10.807	0.363			
Perceived Transparency	PER01	0.799	10.400	0.362	0.851	0.853	0.660
	PER02	0.861	11.519	0.259			
	PER03	0.774	9.979	0.401			

Note: N = 235.

**Table 3:** The mean value, standard deviation, correlation coefficient matrix, and square root of the average of all variables.

Variable	Mean	SD	1	2	3	4
1. Trust	4.655	1.223	0.834			
2. Effectiveness	4.731	0.984	0.699 **	0.774		
3. Discomfort	3.367	1.285	−0.260 **	−0.242 **	0.868	
4. Perceived Transparency	4.748	1.266	0.382 **	0.353 **	0.172 **	0.812

Note: The data on the diagonal line are the square root of the average, and the data on the off-diagonal line are the correlation coefficient between latent variables; ** means *p* < 0.01.

**Table 4:** Model test of mediation analysis.

Dependent Variable	Variable	β	SE	T	95% Confidence Interval		R2	F
					LLCI	ULCI		
Perceived transparency	Constant	4.470 ***	0.116	38.688	4.242	4.697	0.046	11.336 ***
	AI decision-making transparency	0.544 ***	0.162	3.367	0.226	0.863		
Effectiveness	Constant	3.429 ***	0.235	14.596	2.966	3.892	0.125	16.567 ***
	AI decision-making transparency	−0.026	0.124	−0.208	−0.269	0.218		
	Perceived transparency	0.277 ***	0.049	5.662	0.181	0.373		
Discomfort	Constant	2.552 ***	0.323	7.910	1.916	3.188	0.031	3.753 *
	AI decision-making transparency	−0.113	0.170	-0.665	−0.447	0.221		
	Perceived transparency	0.184 **	0.067	2.739	0.052	0.316		
Trust	Constant	0.772 *	0.353	2.189	0.077	1.467	0.531	65.140 ***
	AI decision-making transparency	−0.119	0.113	−1.049	−0.341	0.104		
	Perceived transparency	0.203 ***	0.050	4.084	0.105	0.301		
	Effectiveness	0.734 ***	0.064	11.554	0.609	0.859		
	Discomfort	−0.146 **	0.046	−3.156	−0.237	−0.055		

Note: ① Standardized regression coefficients are reported; ② N = 235; ③ LLCI = lower-level confidence interval, ULCI = upper-level confidence interval; ④ * *p* < 0.05, ** *p* < 0.01, *** *p* < 0.001.

**Table 5:** Mediating effect test.

		Effect	Boot SE	95% Confidence Interval	
				LLCI	ULCI
Total effect		0.086	0.160	−0.229	0.401
Indirect effect		−0.119	0.113	−0.341	0.104
Direct effect	TOTAL	0.204	0.119	−0.027	0.437
	ADT → EPT → ETA	0.111	0.045	0.034	0.212
	ADT → EPE → ETA	−0.019	0.091	−0.199	0.166
	ADT → EDA → ETA	0.017	0.026	−0.033	0.073
	ADT → EPT → EPE → ETA	0.111	0.044	0.037	0.210
	ADT → EPT → EDA → ETA	−0.015	0.009	−0.037	−0.002

Note: ①N=235; ②LLCI = lower-level confidence interval, ULCI = upper-level confidence interval; ③ADT: AI decision-making transparency, EPT: employees’ perceived transparency, EPE: employees’ perceived effectiveness of AI, EDA: employees’ discomfort with AI, ETA: employees’ trust in AI.

## Data Availability

Not applicable.

## Appendix A

In the following, we will describe an application scenario. Please take a moment to read the following scenario carefully. Try to imagine yourself in the situation.

You are an employee of the company Car Solutions (a production company in the car industry). You are a member of the lighting system team, which is responsible for the assembly of headlights. The team is a mixed team consisting of you, four other employees, the team leader and an artificial intelligence (AI) system.

The AI system is responsible for the organization of the team, and its work includes decision-making about task arrangement and task assignment. Thus, the AI system is the decision maker on the team in this area.

Two weeks ago, the AI system informed you that the work process will be restructured for the conversion to a more efficient LED technology. These changes will affect your work and are directly related to you [1].

Ref. [1] AI decision-making non-transparency group is only described here.

Before making a decision, the AI system has to explain to you the rationale for the decision. The AI system collects data about LED lights and then compares the data with the stored data of the original car lights. By calculating various possible effects, the AI system has found that LED car lights are the most effective.

In the decision-making process, the first stage is the goalsetting stage, in which the AI system will explain to you how to set goals and select features. The second stage is the coding stage, in which the AI system reports to you about accuracy, various performance indexes, differences in different samples, when to use what data, training algorithms, and how to clean up. The last stage is the implementation stage, in which the AI system releases the source code and records to you [2].

Ref. [2] AI decision-making transparency group is described here.
